# Clinical and Functional Characterization of a Patient Carrying a Compound Heterozygous Pericentrin Mutation and a Heterozygous IGF1 Receptor Mutation

**DOI:** 10.1371/journal.pone.0038220

**Published:** 2012-05-31

**Authors:** Eva Müller, Desiree Dunstheimer, Jürgen Klammt, Daniela Friebe, Wieland Kiess, Jürgen Kratzsch, Tassilo Kruis, Sandy Laue, Roland Pfäffle, Tillmann Wallborn, Peter H. Heidemann

**Affiliations:** 1 Pediatric Research Center, University Hospital for Children and Adolescents, Leipzig, Germany; 2 Department of Pediatrics, University Hospital for Children and Adolescents, Leipzig, Germany; 3 Institute of Laboratory Medicine and Molecular Diagnostics, Leipzig, Germany; 4 Department of Pediatrics I, Klinikum Augsburg, Augsburg, Germany; Graduate School of Medicine, the University of Tokyo, Japan

## Abstract

Intrauterine and postnatal longitudinal growth is controlled by a strong genetic component that regulates a complex network of endocrine factors integrating them with cellular proliferation, differentiation and apoptotic processes in target tissues, particularly the growth centers of the long bones. Here we report on a patient born small for gestational age (SGA) with severe, proportionate postnatal growth retardation, discreet signs of skeletal dysplasia, microcephaly and moyamoya disease. Initial genetic evaluation revealed a novel heterozygous *IGF1R* p.Leu1361Arg mutation affecting a highly conserved residue with the insulin-like growth factor type 1 receptor suggestive for a disturbance within the somatotropic axis. However, because the mutation did not co-segregate with the phenotype and functional characterization did not reveal an obvious impairment of the ligand depending major IGF1R signaling capabilities a second-site mutation was assumed. Mutational screening of components of the somatotropic axis, constituents of the IGF signaling system and factors involved in cellular proliferation, which are described or suggested to provoke syndromic dwarfism phenotypes, was performed. Two compound heterozygous *PCNT* mutations (p.[Arg585X];[Glu1774X]) were identified leading to the specification of the diagnosis to MOPD II. These investigations underline the need for careful assessment of all available information to derive a firm diagnosis from a sequence aberration.

## Introduction

The process of human growth is an extraordinarily complex system with the somatotropic GH-IGF1 axis in the center of the endocrine regulation of pre- and postnatal growth. Both microcephalic osteodysplastic primordal dwarfism type II (type Majewski or MOPD II, MIM 210720) [1], [2] and mutations in the insulin-like growth factor 1 receptor gene (*IGF1R*, MIM 270450) are very rare causes of pre- and postnatal growth retardation. Starting in 2008 it was discovered that biallelic mutations in the pericentrin gene (*PCNT*) cause MOPD II. Absence of functional PCNT results in disorganized mitotic spindles and missegregation of chromosomes [3]. Mutations in the *IGF1R* gene can result in intrauterine growth retardation (IUGR) without postnatal catch up growth. Aberrant IGF1R expression or protein structure are described to lead to IGF1R haploinsufficiency [4], [5], disturbed processing of the proreceptor [6], [7], decreased ligand binding [8], abrogated IGF1R tyrosine kinase activity and reduced receptor autophosphorylation [9]–[11]. Here we report on a female patient with IUGR and severe postnatal growth failure carrying a novel *IGF1R* mutation and a compound heterozygous mutation in the *PCNT* gene. Additional phenotypic signs were microcephaly, mircodontia, clinodactyly, retarded bone age, and skeletal abnormalities. Due to the initial assumption of an endocrine disturbance underlying the severe growth restriction comprehensive endocrine evaluation was performed but did not reveal any profound abnormalities. To assess the contribution of the IGF1R mutation to the phenotype of the patient *in vitro* studies examining the molecular consequences of the IGF1R mutation were performed.

## Results

### Patient characteristics and genetic analysis

**Figure 1:**
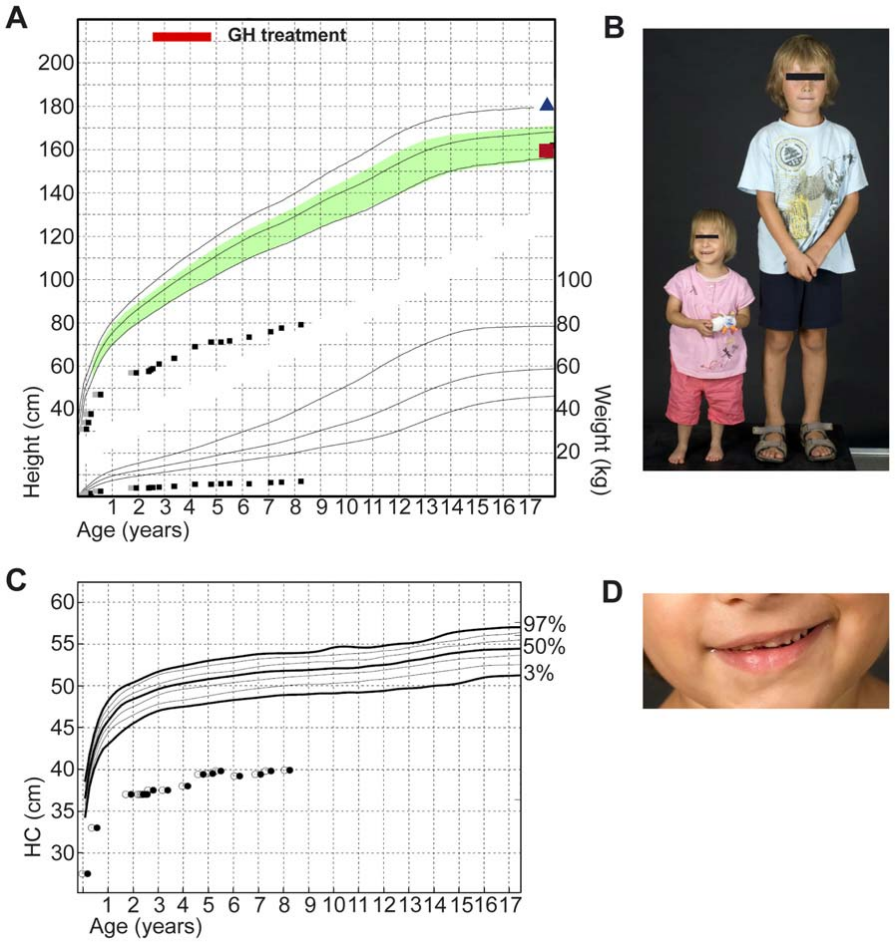
Clinical data of the patient. A, Growth and weight charts of the patient. On the growth chart solid lines indicate 97th, 50th and 3rd percentiles; the shaded green area exhibits target height calculated from parent's height (blue triangle, father's height; red square, mother's height); red bar assigns period of rhGH therapy; grey squares mark the corrected age for preterm infants. B, Photograph of the patient at the age of 5.7 yrs. in comparison to a 5.8 yrs. old boy. C, Head circumference (HC) chart of the patient; 97th, 50th and 3rd percentiles are indicated as black solid lines, white circles mark the corrected age for preterm infants. D, Photograph of the patient's hypoplastic teeth.

**Figure 2:**
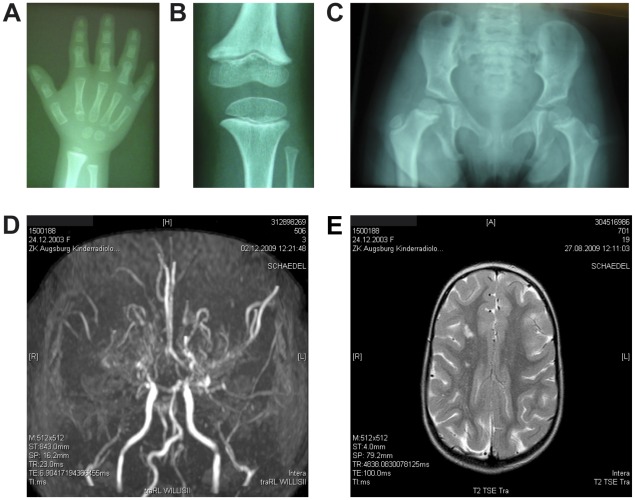
Radiological examinations and neuroimaging. A, Left hand at the age of 4.1 years: mild metaphyseal flaring of distal radius and ulna; shortening of the distal ulna; short metacarpal I; pseudoepiphyses of metacarpals II and V, shortness of the middle and distal phalanges; clinodactyly of the little finger, bone age was retarded (11 months). B, Left knee at the age of 5.7 years: V-shaped flare of the distal femoral metaphyses; triangular shape of the distal femoral epiphyses. C, Hip at the age of 5.7 years: high and narrow pelvis with broad cranial parts of the ischia; irregular metaphyses of the proximal femur; coxa vara. D, Brain MRI at the age of 5.9 years: bilateral occlusion of the intracranial internal carotid artery and the middle cerebral arteries and occlusion of the right anterior cerebral artery, prominent moyamoya collaterals; E, Brain MRI at the age of 5.7 years: old infarcts in the right white matter region.

−The female patient was born with IUGR (height 31.0 cm, -2.0 SDS; weight 680 g, -1.7 SDS corrected for gestational age; HC 21.5, <3^rd^ percentile) after 29 weeks and 6 days of pregnancy as the child of non-consanguineous, Caucasian, healthy parents. The patient's father is of normal height (179.4 cm, -0.256 height-SDS), but her mother was also born small for gestational age (height 47 cm, -1.94 height-SDS; weight 2400 g, -1.9 weight-SDS) and has a final height of 157 cm (-1.75 height-SDS) (**Fig. 1A**). The patient showed no catch-up growth (age 4.8 yr; height 71.2 cm, -8.1 SDS; weight 5.6 kg, -13.1 SDS; BMI 11.0 kg/m^2^, -4.3 SDS; HC 39.4 cm, -7.4 SDS) (**Fig. 1A and 1B**) after birth nor under GH treatment (37,5-69 ug/kg/d) over a period of 27 months. GH has been initiated at the age of 2.5 yr and ended at the age of 4.75 yr because of failure to accelerate growth velocity. Basal GH (0.8 ng/ml) measured at the age of 2.5 yr and GH release stimulated by arginine provocation test (15.8 ng/ml) performed at 1.5 yr showed normal GH secretion. In the course of GH therapy IGF1 serum levels rose steadily from less than -1.9 SDS at several occasions before treatment to 1.1 SDS during GH treatment. A detailed clinical course of the IGF1 serum levels under GH treatment is provided as supplemental material, published as supplemental data on the PLoS ONE website at http://ww.plosone.org (**Fig. S1**). An IGF1 generation test performed at the age of 2.5 years, though, did not result in a substantial increase of IGF1 serum levels (0.15 mg rhGH daily sc; one week IGF1 change from 73 ng/ml to 79 ng/ml). Postnatal growth retardation as well as microcephaly of the girl were profound, but did not worsen over time (**Fig. 1C**). On clinical examination she showed disproportionate shortness of forearms and legs, bilateral clinodactyly V, significant facial asymmetry and hypoplasia of the teeth (**Fig. 1B and 1D**). Radiological skeletal examinations at the age of 4.1 and 5.7 years revealed severely retarded bone age (**Fig. 2A**) and the radiological features of skeletal dysplasia as been described in MOPD II like V-shaped flare of the distal femoral metaphyses with corresponding triangular shape of the distal femoral epiphyses (**Fig. 2B**). The pelvis was high and small and showed the typical coxa vara (**Fig. 2C**) [1], [2].

At the age of 5.7 years she presented with clinical signs of cerebral ischemia like numbness in her right arm and hand. Neuroimaging revealed severe cerebral vascular anomalies classified as moyamoya disease without aneurysms (**Fig. 2D**). In addition old infarcts in the right white matter region could be identified (**Fig. 2E**). Surgical revascularization by encephalomyosynangiosis and encephalodurosynangiosis was performed at the age of 5.9 on the left side and at the age of 6.2 years on the other side to protect against future stroke-like episodes. In addition the patient received an antiplatelet medication. Because she was an acetylsalicyl acid-non-responder the treatment was successfully continued with clopidogrel.

Because of a mild speech delay speech therapy was started between the age of 2 and 3 years. At the age of 7.1 years neuropsychological testing was performed with the Kaufmann Assesment Battery for Children (K-ABC, 2^nd^ edition). The results were more than 2 SD below the mean and spoke in favour of mental retardation.

Since many patients with MOPD II show a high frequency of insulin resistance and diabetes mellitus glucose metabolism was investigated at the age of 7.1 and 7.5 years [3], [12]. Fasting blood glucose was 60 and 70 mg/dl, respectively (normal range: <110 mg/dl) and fasting serum insulin was <2 mU/l (normal range: <2 to 10 mU/l). During an oral glucose tolerance test (1.75 g/kg body weight = 11 g/patient) blood glucose increased from 60 to 71 mg/dl at 120 min (normal reference range: <140 mg/dl) and excluded glucose intolerance at the age of 7.1 years. TSH, free T3, free T4, parathormone, aspartate aminotransferase, alanine aminotransferase, alkaline phosphatase, ferritin, vitamin B12 and folic acid were within the normal range. Neonatal screening for metabolic and endocrine diseases was normal.

The karyotype of the patient was normal (46,XX). Because no precise diagnosis could be obtained from clinical examination molecular genetic testing was performed. dHPLC screening of all coding exons of the *IGF1R* gene and subsequent direct sequencing revealed a novel heterozygous nucleotide transversion from thymine to guanine in exon 21 (c.4082T>G). This mutation leads to an amino acid exchange from leucine to arginine in position 1361 of the IGF1R protein (incl. signal peptide; p.L1361R). Subsequently, all coding exons of the *IGF1R* gene were analyzed and no further sequence aberration was detected. Sequence analysis of exon 21 of 50 unaffected individuals did not show any sequence aberration at position 4082. The non-conservative amino acid substitution affects a residue that is highly conserved among different species (**Tab. 1**) and is predicted to be probably damaging (PolyPhen v2; http://genetics.bwh.harvard.edu/pph2/). The patient's father was identified as carrier of the IGF1R-L1361R mutation, whereas exon 21 of the mother was normal. So far, IGF1R mutations are described to be inherited in a dominant way. Due to the apparent lack of co-segregation of the mutant IGF1R-L1361R allele with the growth retarded phenotype and the *in vitro* data presented below, the existence of a second-site mutation was hypothesized, which alone or in interplay with the IGF1R mutation might cause the extraordinary phenotype of the patient. Genetic analysis of other candidate genes including those of the somatotropic axis [*IGF1*, growth hormone (*GH1*), GH receptor (*GHR*), GH releasing hormone (*GHRH*), and GHRH receptor (*GHRHR*)], and genes of the IGF1R signaling system [insulin receptor (*INSR*), insulin receptor substrate 1 (*IRS1*) and GAIP C-terminus-interacting protein 1, synectin (*GIPC1*)] yielded no conspicuous findings. Sequencing of the *STAT5B* gene identified a heterozygous polymorphism 38bp upstream of exon 6 but was assumed to be not related to the clinical manifestations of the patient. DNA diagnostics excluded uniparental disomy of chromosome 14 [upd(14)mat) [13], chromosome 2 and 16 (upd(2)mat; upd(16)mat [14]], and chromosome 7 (Silver-Russell syndrome, MIM 180860). Analysis of chromosome 15 showed a normal methylation pattern and no suspicion of Prader-Willi syndrome (MIM 176270).

**Table 1:** Sequence comparison of the IGF1R carboxyl terminus among different species.

	1330	1340	1350	1360
	|	|	|	|
IGF1R human (patient)*	GPGVLVLRAS	FDERQPYAHM	NGGRKNERALP	RPQSSTC
IGF1R human (wild type)	GPGVLVLRAS	FDERQPYAHM	NGGRKNERALP	LPQSSTC
IGF1R mouse	GPGVLVLRAS	FDERQPYAHM	NGGRANERALP	LPQSSTC
IGF1R bovine	GPGVLVLRAS	FDERQPYAHM	NGGRKNERALP	LPQSSTC
IGF1R X.laevis	GPGVVVLRAS	FDERQPYAHM	NGGRKNERALP	LPQSSAC
Consensus	GPGVLVLRAS	FDERQPYAHM	NGGRKNERALP	LPQSSTC

* ) amino acid numbering according to human UniProtKB acc. P08069; position of the patient's mutation is marked in bold.

However, two heterozygous mutations in the *PCNT* gene [c.1753C>T (p.R585X) and c.5320G>T (p.E1774X)] were identified. These mutations lead to premature stop codons like in other patients with MOPD II [3], [15]. The mother was heterozygous carrier for the c.5320G>T nonsense mutation and the father heterozygous carrier for the c.1753C>T nonsense mutation confirming compound heterozygosity in the affected patient. Consequently, the diagnosis has been specified to MOPD II, which is described to be a genetically homogeneous condition due to loss-of-function of PCNT [15].

### Cell surface expression and IGF1 induced phosphorylation of IGF1R, Akt and Mapk/Erk

Although the IGF1R-L1361R mutation does not map to major structural domains within the receptor known to be involved in IGF1 ligand binding or kinase activity, we analyzed the signal transduction capabilities of the mutant receptor with respect to IGF1 induced receptor autophosphorylation and activation of major downstream signaling molecules.

**Figure 3:**
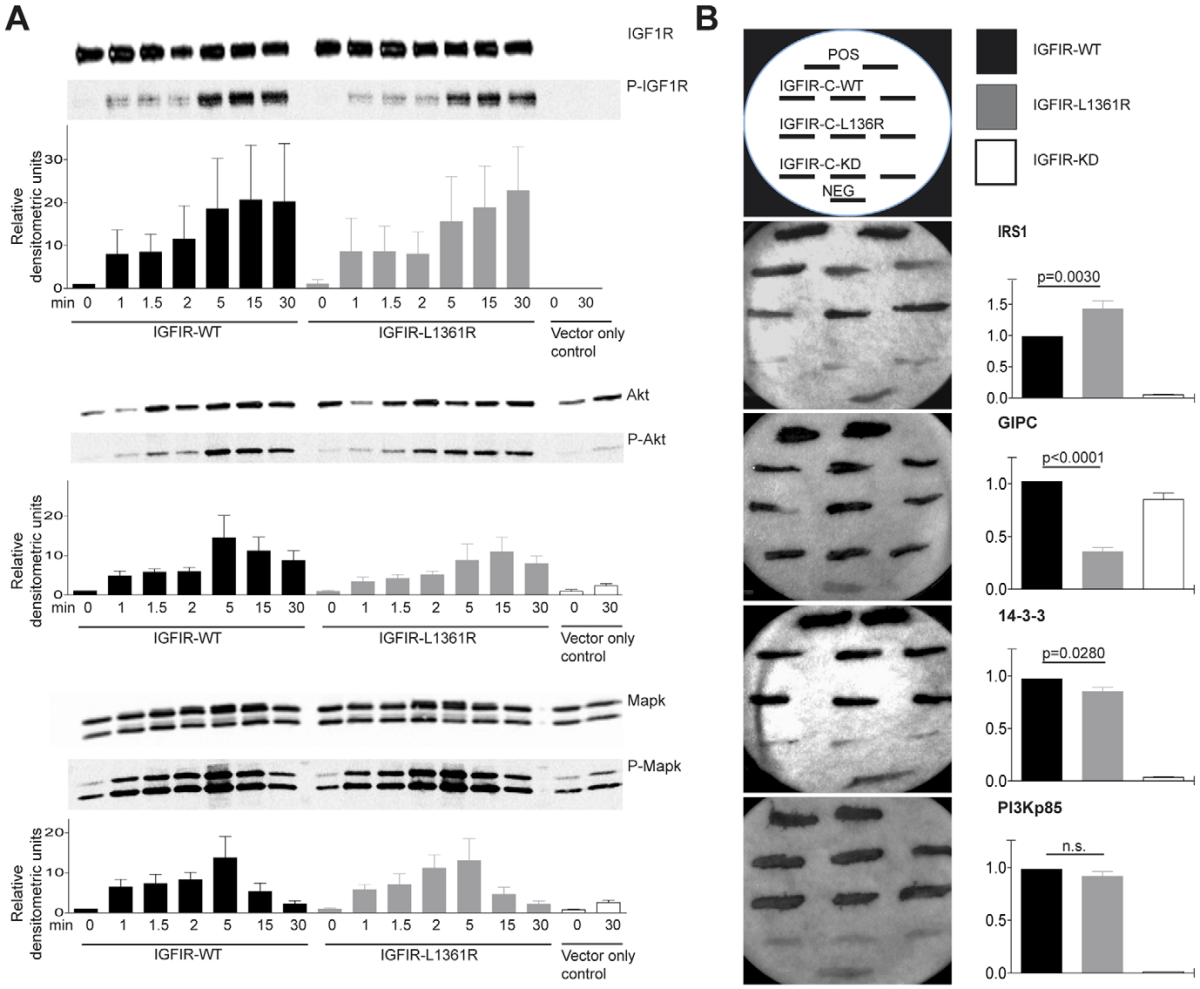
IGF1 induced IGF1R phosphorylation and downstream signaling. A, Wild type and L1361R mutant IGF1R autophosphorylation in transiently transfected R^−^ cells after stimulation with 10 nM IGF1 for 0–30 minutes. Immunoblots were incubated with specific antibodies against phosphorylated IGF1R β-subunit (P-IGF1R), Akt (P-Akt) or Mapk/Erk (P-Mapk/Erk), stripped and subsequently incubated with specific antibodies against the IGF1R α-subunit (IGF1R), Akt (Akt), or Mapk/Erk (Mapk/Erk). IGF1R α-subunit levels were assessed as control of successful transfection. Densitometric units were normalized to total levels of IGF1R α-subunit and the fold increase was calculated (IGF1R-WT at 0 min was set 1). Results are shown as means ± SEM calculated from four independent experiments. B, Yeast two-hybrid analysis of the interaction of the IGF1R derivatives (IGF1R-C-WT, IGF1R-C-L1361R, IGF1R-C-KD) with adapter proteins (IRS1, 14-3-3ß, GIPC, PI3Kp85) by lift-off assay (left panel). Positive interactions led to blue staining (here black) of the colonies. POS, positive control p53 x large T-antigen; NEG, negative control Lamin x large T-antigen. The filter assays shown are representative for more than three independent experiments. Quantification of protein-protein interaction was measured using ONPG-assays and data are shown as mean ± SEM (right panel).

Stimulation of the patient's and wild type fibroblasts with 13 nM IGF1 resulted in a comparable phosphorylation of the IGF1R in both fibroblast cultures, while incubation without IGF1 showed, as expected, no IGF1R autophosphorylation. Immunoblotting of unphosphorylated IGF1R revealed equal expression of the IGF1R in mutant and control cells. Moreover, undisturbed receptor expression at the cell surface was confirmed by flow cytometry. See supplemental material on PLoS ONE Online website at http://www.plosone.org (**Fig. S2**). To investigate the dose and time dependent effects of IGF1 on the activation of the mutant receptor without masking by the endogenous wild type allele, we then determined IGF1 induced IGF1R phosphorylation and activation of its downstream targets Akt and Mapk/Erk in R^−^ cells transfected with IGF1R-WT, IGF1R-L1361R and pcDNA3+ (vector control). Phosphorylation was analyzed at different time points and different concentrations of IGF1 as outlined in Materials and Methods. IGF1R-WT and IGF1R-L1361R transfected R^−^ cells revealed a time dependent increase in the phosphorylation of the β-subunit of IGF1R, and the Akt and Mapk/Erk downstream effectors in the presence of IGF1 (**Fig. 3A**). Also, we detected a dose dependent activation of IGF1R, Akt and Erk (data not shown). However, neither receptor autophosphorylation nor downstream signaling was found to be different in IGF1R-WT and IGF1R-L1361R transfected R^−^ cells.

Thus, the heterozygous mutation at the carboxy-terminus of the IGF1R appears not to affect the IGF1 induced downstream signaling of the two tested major IGF1R pathways (**Fig. 3A**).

### Protein-protein interaction of the mutant IGF1R with adapter proteins

The IGF1R binds several adapter proteins at different docking sites, of which some are located within the C-terminal tail of the receptor and, thus, reside near to our identified mutation. Some of these interacting molecules (14-3-3β, GIPC) are involved in IGF1R signal transduction without directly affecting the previously analyzed MAPK and AKT pathways. Therefore, we investigated whether the L1361R mutation affects protein-protein interactions and quantified them in a yeast two-hybrid system (**Fig. 3B**).

Co-transformation of yeast cells with wild type IGF1R-C and IRS1, p85PI3-K, 14-3-3β, or GIPC resulted in a rapid blue staining after incubation with X-Gal. The IGF1R-C-K1003A mutant does not exhibit kinase activity and therefore kinase dependent interactions of IRS1, p85PI3, 14-3-3ß are abolished. Interaction with GIPC has been shown to be kinase independent [16]. As expected, using this negative control lift-off assays of IRS1, p85PI3-K and 14-3-3ß yielded no ß-galactosidase activity of yeast colonies. In contrast, interaction of IGF1R-C-K1003A with GIPC was unaffected and resulted in blue color formation. Lift-off filter assays of mutant IGF1R-C-L1361R confirmed β-galactosidase reporter gene activation for all tested adapter proteins by detection of blue stained colonies. To verify and quantify two-hybrid interactions liquid cultures assays for β-galactosidase activity using ONPG as substrate were performed. The ability of the positive control IRS1 to associate with IGF1R-C-L1361R was moderately but significantly increased compared to wild type IGF1R-C. Co-transformation of IGF1R-C-L1361R with 14-3-3ß demonstrated a modest decrease in reporter activation. Interaction of p85PI3-K with IGF1R-C-L1361R was not altered compared to wild type IGF1R-C. Contrary, GIPC, which has been reported to interact specifically with the COOH-terminal amino acid 1357–1367 of the IGF1R but not the insulin receptor [16], demonstrated an approximately 50% decrease in interaction with the IGF1R-C-L1361R.

### Proliferation of patient's fibroblasts

**Figure 4:**
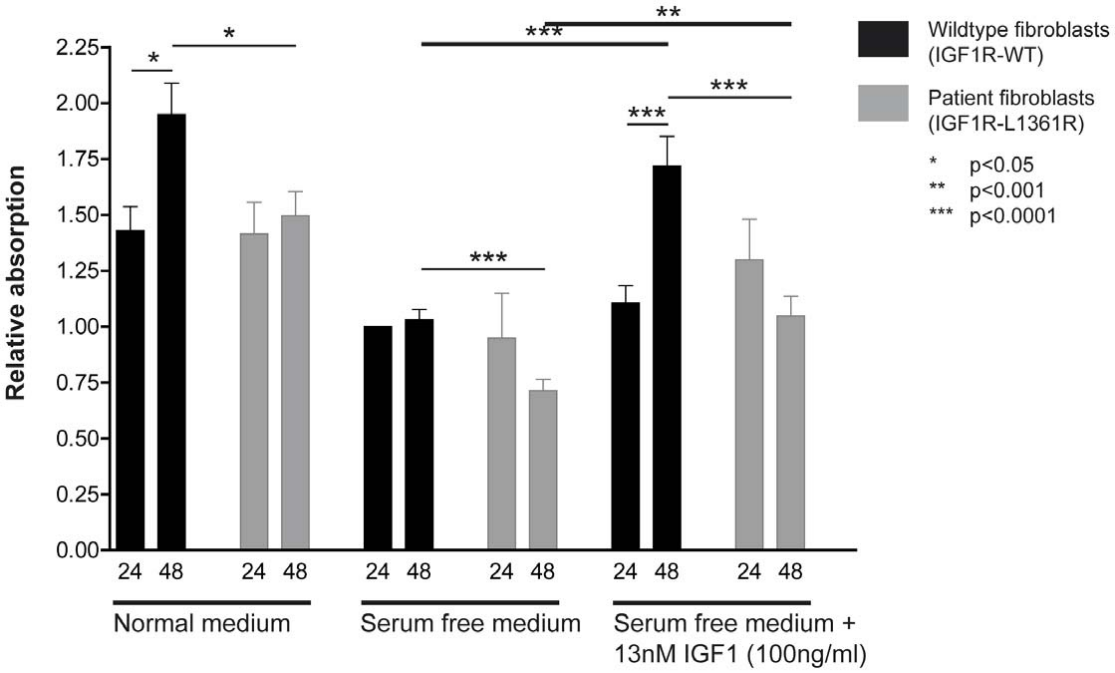
Proliferation of patient's and wild type fibroblasts. Proliferation of patient's fibroblasts (grey bars) and wild type fibroblasts (black bars) as measured with WST-1 reagent assay. Cells were cultured for 24 h and 48 h in normal culture medium (NM), serum free medium (SFM), serum free medium supplemented with 13 nM (100 ng/ml) IGF1 (SFM+IGF1). Then WST-1 reagent was added and absorbance was measured. Data were normalized to the absorbance value obtained for wild type fibroblasts cultured in SFM for 24 h. Proliferation was significantly measurable in wild type fibroblasts in NM, SFM, SFM+IGF1 between 24 h and 48 h. In contrast, there was no significant increase between 24 h and 48 h found in patient fibroblasts cultured in NM, SFM, SFM+IGF1. Results were calculated from more than three independent experiments as mean of the relative absorbance with ± SEM.

We next investigated the proliferation capacity of the patient's fibroblasts compared to wild type fibroblasts. We found no difference in proliferation rate between wild type and patient's fibroblasts within 24 h in normal culture medium, serum free medium and serum free medium supplemented with IGF1. After additional 24 h incubation the wild type fibroblasts proliferated significantly in normal culture medium and serum free medium supplemented with IGF1. In contrast, we detected no further proliferation of the patient's fibroblasts after additional 24 h incubation. Hence, patient fibroblasts exhibited a significantly decreased cell proliferation capacity despite an early response to IGF1 (**Fig. 4**).

## Discussion

Herein we report a preterm girl with IUGR, primordial dwarfism, microcephaly, discreet skeletal dysplasia, and mild facial dysmorphism during the newborn period and infancy.

Laboratory measurements were not indicative for pituitary insufficiency or GH insensitivity, however, IGF1 was low. Owing to the unclear etiology of the patient's condition a comprehensive genetic evaluation was initiated. Analysis of the *IGF1R* gene revealed a novel heterozygous variation (p.Leu1361Arg) that was subjected to further molecular characterization to assess its biochemical properties and cell-physiological effects. However, due to the incomplete compatibility of the identified IGF1R variant with the established features of IGF1R mutation triggered IGF1 resistance (foremost the lack of co-segregation of the usually dominantly inherited growth restriction with the IGF1R mutation) genetic testing of additional candidate genes was continued. The rationale behind these efforts was to evaluate the possibility of the co-existence of defects in two or more functionally interacting proteins that do not follow obvious Mendelian inheritance patterns. Such oligo- or digenic action of interacting alleles of distinct genes becomes – if mutated – increasingly recognized as a mode of disease transmission in the continuum between Mendelian and complex traits [17]. Genetic testing comprised components of the somatotropic axis, constituents of the IGF signaling system and factors involved in cellular proliferation, which are described or suggested to provoke syndromic or nonsyndromic dwarfism phenotypes. As a major finding two compound heterozygous nonsense mutations within the *PCNT* gene were identified (p.[Arg585X];[Glu1774X]), which are suggested to cause the patient's phenotype. As a consequence, the diagnosis was specified as MOPD II. The mutation [(c.1753C>T(p.Arg585X)], inherited by the father, was already described by Willems et al. [15]. The second mutation, inherited by the mother, [(c.5320G>T (p.Glu1774X)] is a novel defect to our knowledge. Both mutations lead to premature stop codons like in other patients with MOPD II [3], [15].

The *PCNT* gene encodes the centrosome protein pericentrin which organizes the mitotic spindle for segregation of the chromosomes during cell division and influences the cell cycle progression. Rauch et al suggested that mitotic centrosome dysfunction results in loss of cellularity, cell deaths and growth restriction [3]. Thus pericentrin mutations can be expected to cause disturbances in cell division and finally in growth of the body and brain. MOPD II is a rare syndrome of extreme intrauterine and postnatal growth retardation, microcephaly, resistance to growth hormone, severe insulin resistance, bone and dental dysplasia. Although most newborns born small for gestational age show spontaneous catch-up growth within two to four years of life, this does not happen in MOPD II. In addition, MOPD II patients appear to have a resistance to growth hormone. Huang-Doran et al described two patients in whom growth hormone therapy was stopped because of inefficacy [12]. Our patient did not show any catch-up growth during growth hormone therapy though the dose was titrated to 69 ug/kg/day and IGF 1 increased into the upper normal range.

Defects in pericentrin are associated with severe insulin resistance and diabetes mellitus [12]. 17 out of 21 patients had insulin resistance proven by elevated fasting insulin concentrations and 10 out of 21 patients had early onset diabetes mellitus (mean age 15 years with a range of 5 to 28). All patients without insulin resistance were younger than four years. As an exception of the rule glucose tolerance was still normal in our patient at the age of 7.1 years.

In spite of microcephaly brain development appears to be grossly normal and most patients are said to have mild mental retardation [3], [15]. However, results of standardized examinations are lacking in the literature. In our patient cognitive development was more reduced than expected from a clinical point of view.

In patients with MOPD II life expectancy is reduced by stroke like episodes and intracranial hemorrhages secondary to moyamoya disease like cerebrovascular anomalies and aneurysms.

A review and natural history study by Hall et al. [18] documented cerebral aneurysms or moyamoya angiopathy in 11 of 58 (19%) patients, leading to at least four deaths. A clinical report and review by Brancati et al. [19] documented that 15 of 63 (24%) patients were found to have cerebral aneurysms or moyamoya disease. 25 patients with the diagnosis of MOPD II were followed by an Institutional Review Board (IRB) [20]. In this registry a higher number of 52% (13 of 25) have been found to have cerebral neurovascular abnormalities including moyamoya angiopathy and/or intracranial aneurysms. The increased incidence of cerebrovascular disease in this cohort might be based upon the used MRI/MRA screening which detected abnormalities also in asymptomatic individuals. Observed cerebral vascular anomalies include moyamoya disease or multiple aneurysms. There is only one report of a patient with both moyamoya disease and multiple aneurysms by Waldron et al. [21]. Patients with moyamoya disease show an earlier age at onset of these complications compared to the group with intracranial aneurysms. Although this latter subset of subjects had worse prognosis [22], [23]. The occurrence of cerebral vascular anomalies already at an early age highlights the importance of a timely neuroimaging in the clinical management of these patients.

In some patients moyamoya disease was associated with cutis marmorata [19], [24], but not in our patient. It remains unclear how the underlying genetic change leads to moyamoya angiopathy. We agree that screening for moyamoya disease at the time of MOPD II diagnosis and at least every 12–18 months, as suggested by the IRB, should be performed to identify and treat progressive and life threating cerebrovascular disease. If diagnosed early enough, re-vascularization and aneurysm treatment in skilled hands can be performed safely and might prevent or minimize long-term sequelae in this population like in our patient so far.

Several mutations in the IGF1R have been reported to result in common phenotypic features like IUGR, stunted postnatal growth development, and microcephaly [4]–[11], [25]. Generally, growth retardation due to heterozygous IGF1R mutations has been observed to occur in the borderline range (around -2 SDS) but can exceed these limits down to nearly -6 SDS if non-genetic factors or the genetic background are unfavorable [8], [9]. Growth deficit may even worsen if both IGF1R alleles are affected resulting in height of -7.3 SDS at the age of 3 years [25]. Moreover, complete receptor loss due to targeted disruption of the *Igf1r* gene in mice leads to severely growth retarded offspring showing a birth length of 45% compared to wild type littermates [26]. Although, in our patient the profound growth deficit certainly is caused by the aberrant pericentrin genes/proteins, such findings suggest that dwarfism-like phenotypes can also be provoked by IGF1R mutations under specific, adverse genetic or environmental conditions. Moreover, the impact of co-occurring variants in additional gene/s either residing on the same or unrelated growth regulating pathways has not yet been described for the IGF1R.

Initial genetic analysis revealed a novel heterozygous p.L1361R mutation within the IGF1R. Although this non-conservative amino acid substitution affects a highly phylogenetically conserved residue at the very COOH-terminal end of the IGF1R there is a remarkable lack of co-segregation of the mutation with the growth retarded phenotype within the family. Moreover, IGF1 receptor mutations basically manifest as hormone resistance characterized by normal or elevated IGF1 serum concentrations. In contrast, the index patient displayed low-normal to decreased IGF1 levels at several occasions before GH therapy. Although the decrease might indicate some undefined additional IGF deficiency, sequencing of the IGF1 gene as well as pituitary function tests and IGF1 response during GH therapy were normal. It appears likely, that reduced IGF1 levels might rather be a reflection of severe IUGR without catch-up growth and low BMI [27]. Noteworthy, the endocrine state in patients with *PCNT* mutations has not been evaluated systematically, but IGF1 levels in Seckel and MOPD II patients have been reported occasionally to be normal [28], [29], elevated [30], [31] or low [28], [30], [32].

To elucidate the impact of the p.L1361R mutation and because the function of the COOH-terminal tail of the IGF1R is only poorly defined and no human mutation in this receptor portion has been identified so far [33] we performed comprehensive functional analysis.

Our and other groups showed that IGF1R mutations led to disturbances of receptor trafficking [7] and abrogation of the IGF1R tyrosine kinase activity [10], [11]. Investigations of cell surface expression, cellular expression and IGF1 dependent phosphorylation of the mutant IGF1R were found to be normal as was the activation of major signaling molecules. Since activation was not modified we then addressed the question whether the interactions of the mutant IGF1R with downstream signaling proteins known to associate with the COOH-terminal tail of the receptor (GIPC, 14-3-3, PI3Kp85) are affected. We found an unchanged interaction between the mutant IGF1 receptor and the PI3Kp85 protein and a significantly although moderately decreased association with 14-3-3ß. In addition, association of the IRS1 positive control with the mutant IGF1R was slightly enhanced. It is described in literature that mutations and deletion of the carboxy-terminal tail of the IGF1 receptor result in a higher affinity of the receptor to IRS1 [34]. Most interestingly, we detected an approximately 50% decrease in interaction between the mutant receptor and the GIPC protein. Interaction of GIPC with the IGF1R was first established by Ligensa et al. using a yeast two-hybrid approach and has been shown to act positively in IGF1 receptor signal transduction in *Xenopus* oocytes [16], [35]. Increased expression and activation of the IGF1R and its downstream signaling targets are related to human cancers [36], [37]. Muders et al. showed that GIPC expression is increased in human cells of pancreatic adenocarcinoma (PCA) and knockdown of GIPC results in decreased proliferation of PCA cells [38], [39]. It was hypothesized that GIPC is important for IGF1R membrane stabilization [40], IGF1R trafficking and prevention of IGF1R degradation [41]. Following studies showed that GIPC is involved in IGF1 induced proliferation of different cancer cell lines and cancer cell survival [42]. However, seeking for a ‘second site’ mutation within the possibly epistatically interacting *GIPC1* gene was without finding. Therefore, we suggest that the impaired interaction of GIPC and the mutant IGF1R might elicit subtle physiological effects that are neither obviously associated with human growth control nor easily detectable in the fibroblast assays as performed in our investigations. Additional experiments should be initiated to assess the physiological relevance of this altered interaction.

Cell proliferation of patient fibroblasts was decreased compared to wild type cells and IGF1 application was only in the short term (24 h) able to ameliorate the deficit. Failure to stimulate cell division in response to IGF1 is reminiscent of the absent success of rhGH therapy in the patient that to a large part relies on the stimulation of endogenous IGF1 expression and action. However, care must be taken if deducing the *in vivo* situation from *in vitro* data obtained from fibroblast studies. The identified compound heterozygous *PCNT* mutations may provide a plausible explanation for the observed reduced cell proliferation and therefore the patient's phenotype. Several mechanisms have been suggested that link pericentrin mutations with the dramatically reduced body size in mice and humans. In all models disruption of the multifunctional scaffolding properties of pericentrin has been suggested to result in a massive loss of cellularity due to centrosome dysfunction [43].

In conclusion, although the assumption of a second-site mutation led to the identification of the pericentrin mutations it is unlikely that the IGF1R mutation contributes to the patient's phenotype. Clinical manifestation of the unaffected father as carrier of the *PCNT* p.R585X loss-of-function mutation and the p.L1361R IGF1R – if at all – hypomorphic mutation as well as demonstration that the major signaling pathways induced by the mutant IGF1R are largely unimpaired suggest that the compound heterozygous *PCNT* mutations on its own represent the underlying cause of the patient's phenotype.

Nonetheless, the quest for the ‘second hit’ remains a promising challenge in polygenical traits such as growth, specifically in light of the high portion of growth retarded, IGF1 resistant children with unknown etiology and the considerable number of unique or rare – possibly hypomorphic – allelic variants in the *IGF1R* gene (unpublished data from our laboratory).

Moreover, our comprehensive work underlines the importance of investigation of the functional relevance of mutations found by diagnostic screenings to assess their pathogenic impact and for a better understanding of the differential clinical picture possibly affecting subsequent therapy strategies. Molecular biological verification of identified mutations should be considered and careful integration with clinical and genetic data is required to avoid missing so far unidentified causative mechanisms.

In summary, we have identified a severely growth retarded girl carrying a non-conservative amino acid exchange in the COOH-terminal tail of the IGF1R. The functional analysis of the IGF1R mutation revealed that the mutation might have subtle effects on IGF1R biology but is assumed not to be causally related to the patient's phenotype. We suggest that the *PCNT* mutations account for the severe growth retardation and microcephaly due to severly diminished cell proliferation.

## Materials and Methods

All investigations were performed after approval of the Ethical Committee of the Medical Faculty of the University of Leipzig (project-no.: 234-2006, date of report: 16.02.2007) and written informed consent of the patient's parents for molecular analysis and publication as well as written informed consent of the same age boy's parents for publication.

### Genetic analysis and endocrine evaluation

All coding exons of the *IGF1R* (NM_000875.3), *IGF1*, growth hormone (*GH1*), GH receptor (*GHR*), GH releasing hormone (*GHRH*), and GHRH receptor (*GHRHR*) genes were amplified by PCR from peripheral blood DNA. Screening for sequence aberrations was performed by temperature modulated heteroduplex HPLC on a reverse-phase column using the WAVE-System (Transgenomics, Crewe, UK). PCR products displaying aberrant chromatograms were further analyzed by direct sequencing. Additional re-sequencing analyses [insulin receptor (*INSR*), insulin receptor substrate 1 (*IRS1*), GAIP C-terminus-interacting protein 1 (synectin, *GIPC1*), signal-transducer and activator of transcription 5B (*STAT5B*), pericentrin (*PCNT*; NM_006031.5)] were performed by direct sequencing of exon-specific genomic PCR fragments without dHPLC pre-screening. Primer sequences can be obtained upon request. DNA tests to investigate uniparental disomies were performed by PCR using locus specific microsatellite markers. IGF1 levels were measured with Immulite 2000 (analytical sensitivity 20 ng/ml, intraassay CV 3.05%, interassay CV 6.16%; Siemens, Munich, Germany). All other hormones were analyzed at the hospital laboratories of Leipzig and Augsburg by certified standard techniques.

### Plasmid construction

IGF1R wild type cDNA was kindly provided by Dr. R. Furlanetto (previously Medical University of South Carolina, SC) and cloned into a pBSK+ plasmid. The IGF1R mutation was introduced into the wild type cDNA by PCR using a mutated adapter primer. The IGF1R-L1361R PCR fragment was ligated into the pBSK+IGF1R plasmid and thereafter subcloned either into the expression plasmid pcDNA3-IGF1R (pcDNA3-IGF1R-L1361R) or into pLexA-IGF1R-C for yeast-two-hybrid assays (pLexA-IGF1R-L1361R). The latter one encodes for a fusion protein of the intracellular domain of the IGF1R (IGF1R-C) and a LexA DNA binding domain.

The construction of “kinase-dead” mutant pcDNA3-IGF1R(K1003A) was performed as previously described [10]. Construction of plasmids encoding for fusion proteins comprising the transcription activation domain B42 (B42-AD) and IRS1, p85PI3-K, or 14-3-3β was previously described [44]. The human GIPC cDNA (*GIPC1*, synectin, MIM 605072) was cloned by RT-PCR using RNA isolated from the MCF-7 breast cancer cell line as template. Detailed description of all cloning procedures can be provided upon request.

### Cell culture and transient transfection

Patient fibroblasts were cultured from a forearm skin biopsy. Wild type human control fibroblasts of age- and sex-matched donors were purchased from the American Type Culture Collection (LGC Standards, Wesel, Germany). Human fibroblasts and *Igf1r* deficient (R^−^) fibroblasts were cultured under standart conditions.

For transient transfection R^−^ cells were grown to confluence and transfected with empty pcDNA3.1+, wild type pcDNA3-IGF1R or pcDNA3-IGF1-L1361R using Lipofectamine2000 (Invitrogen, Karlsruhe, Germany) as described in the manufacturer's manual.

### Flow cytometry

IGF1R cell surface expression of patient and control fibroblasts were analyzed as previously described [7] using flow cytometry (Epics XL, Beckman Coulter, Miami, FL). Fibroblasts were stained with phycoerythrin-conjugated human IGF1R mAb (R&D Systems, Minneapolis, MN). An appropriate isotype control (R&D Systems) was applied for gating.

### IGF1 stimulated phosphorylation of the IGF1R

Expression of unphosphorylated and phosphorylated IGF1R protein was determined in patient and wild type fibroblasts. Therefore, the cells were seeded into 175 cm^2^ flasks in culture medium, kept in serum free medium containing 0.2% BSA (Invitrogen) overnight and then stimulated with 13 nM (100 ng/ml) IGF1 (Amersham Pharmacia Biotech, Uppsala, Sweden) for 15 min at 37°C.

R^−^ cells were used for measuring time and dose dependent IGF1 induced phosphorylation of IGF1R, Akt and Mapk/Erk. 24 hours after transfection cells were starved overnight in serum free medium containing 0.2% BSA (Invitrogen). To detect dose-dependent activation of the IGF1R, cells were incubated with increasing amounts of IGF1 in serum free media (0, 0.1, 1, 3, 5, 10, 100 nM) for 15 min at 37°C. To detect time-dependent activation of the IGF1R, cells were incubated with 10 nM IGF1 in serum free medium for 0, 1, 1.5, 2, 5, 15, 30 minutes. Cell lysis was performed as previously described [7] and lysates were used for immunoblotting.

### Immunoblotting

Expression and ligand-induced phosphorylation of IGF1R, Akt, and Mapk/Erk were measured by immunoblotting using specific antibodies. Preparation of whole-cell lysates and subsequent immunoblotting were performed as described before [7]. Anti-phospho-Tyr^1135/1136^-IGF1Rß #3024, anti-phospho-Ser^473^-Akt antibody #9271, anti-phospho-Thr^202^/Tyr^204^-p44/42 Mapk/Erk1/2 #9101, anti-Akt #9272, anti-p44/42 Mapk/Erk1/2 #9102 (New England Biolabs, Frankfurt/Main, Germany), anti-IGF1Rα #N-20 (Santa Cruz Biotechnology, Heidelberg, Germany) and anti-ß-actin monoclonal antibody (Sigma-Aldrich, Schnelldorf, Germany) were applied as primary antibodies. Horseradish peroxidase conjugated goat anti-rabbit antibody (Thermo Fisher Scientific, Bonn, Germany) and goat anti-mouse antibody (Dako, Hamburg, Germany) were used as secondary antibodies.

### Yeast-two-hybrid assays

EGY48/LacZ yeast strain was used for co-transformation with pLexA and pB42 constructs applying polyethylene glycol and lithium acetate. All yeast incubations were performed at 30°C. Colony-lift filter assays and liquid culture assays using OPNG (o-nitrophenyl β-D-galactopyranoside; Sigma-Aldrich) were performed according to Clontech's Matchmaker protocol (Clontech BD Biosciences, Palo Alto, CA). Absorption measurements for liquid culture assays were performed at 420 nm and β-galactosidase units were calculated according to the Miller formula.

### Proliferation assay

Proliferative capacity of the patient's and wild type fibroblasts was assessed using Cell Proliferation Reagent WST-1 (Roche Diagnostics, Mannheim, Germany). The cells were allowed to grow for 24 h or 48 h in culture medium, serum free culture medium or serum free culture medium supplemented with 13 nM (100 ng/ml) IGF1. WST-1 reagent was added followed by incubation at 37°C for 2 h. Absorption was measured at 440 nm.

## Supporting Information

Figure S1 **Clinical course of the IGF1 serum levels before and under GH treatment.** In the course of GH therapy IGF1 serum levels rose steadily from less than -1.9 SDS at several occasions before GH treatment to 1.1 SDS under GH treatment. Black bar assigns period of rhGH therapy.
(TIF)

Figure S2 **Protein and cell surface expression of IGF1R in wild type and patient's fibroblasts.** A, Protein expression and autophosphorylation of IGF1R of wild type fibroblasts (IGF1R-WT), patient's fibroblasts (IGF1R-L1361R) and parental fibroblasts after stimulation with 13 nM (100 ng/ml) IGF1 for 15 minutes was assessed by immunoblotting. Blots were incubated with specific antibodies against phosphorylated IGF1R β-subunit (P-IGF1R), stripped and subsequently incubated with specific antibodies against IGF1R α-subunit (IGF1R). Immunoblots shown are representative for three independent experiments. B, Expression of the IGF1R on cell surface of wild type and patient's fibroblasts. Cells were stained with phycoerythrin (PE) labelled antibodies and analyzed by flow cytometry. Black curve marks cells labeled with isotype control PE antibody, white curve marks cells labeled with human anti-IGF1R-PE antibody. Percentages of IGF1R PE positive cells are indicated. The mean fluorescence intensity of the IGF1R-phycoerythrin-antibody positive cells normalized to wild type fibroblasts represent the amount of cell surface IGF1R. Results are shown as means ± SEM calculated from more than three independent experiments.
(TIF)
