# Supplementary figures and images for: Clinical and Functional Characterization of a Patient Carrying a Compound Heterozygous Pericentrin Mutation and a Heterozygous IGF1 Receptor Mutation
Source: PLoS One. 2012 May 31;7(5):e38220. doi: 10.1371/journal.pone.0038220 (PMC3365032; doi:10.1371/journal.pone.0038220)

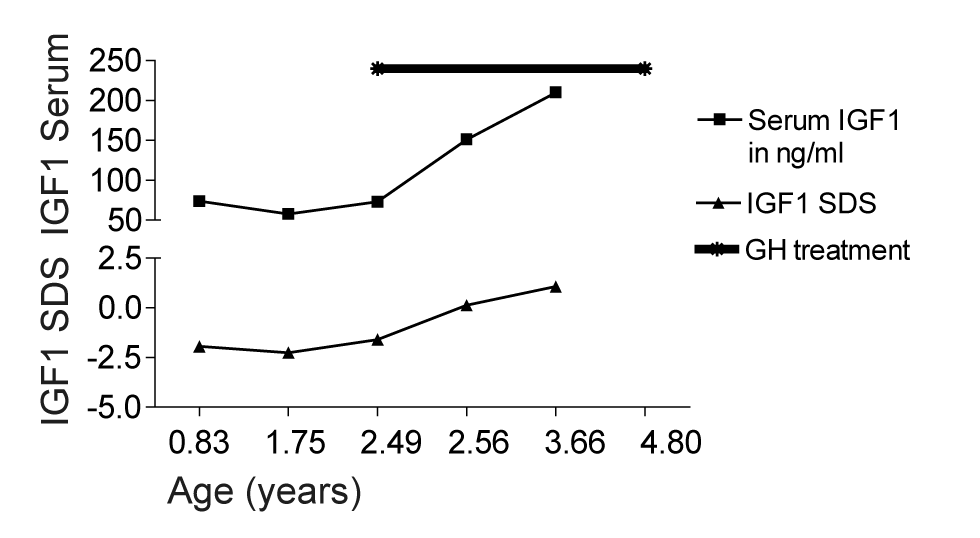

Supplement: Figure S1 — Clinical course of the IGF1 serum levels before and under GH treatment. In the course of GH therapy IGF1 serum levels rose steadily from less than -1.9 SDS at several occasions before GH treatment to 1.1 SDS under GH treatment. Black bar assigns period of rhGH therapy. (TIF) [file pone.0038220.s001.tif]

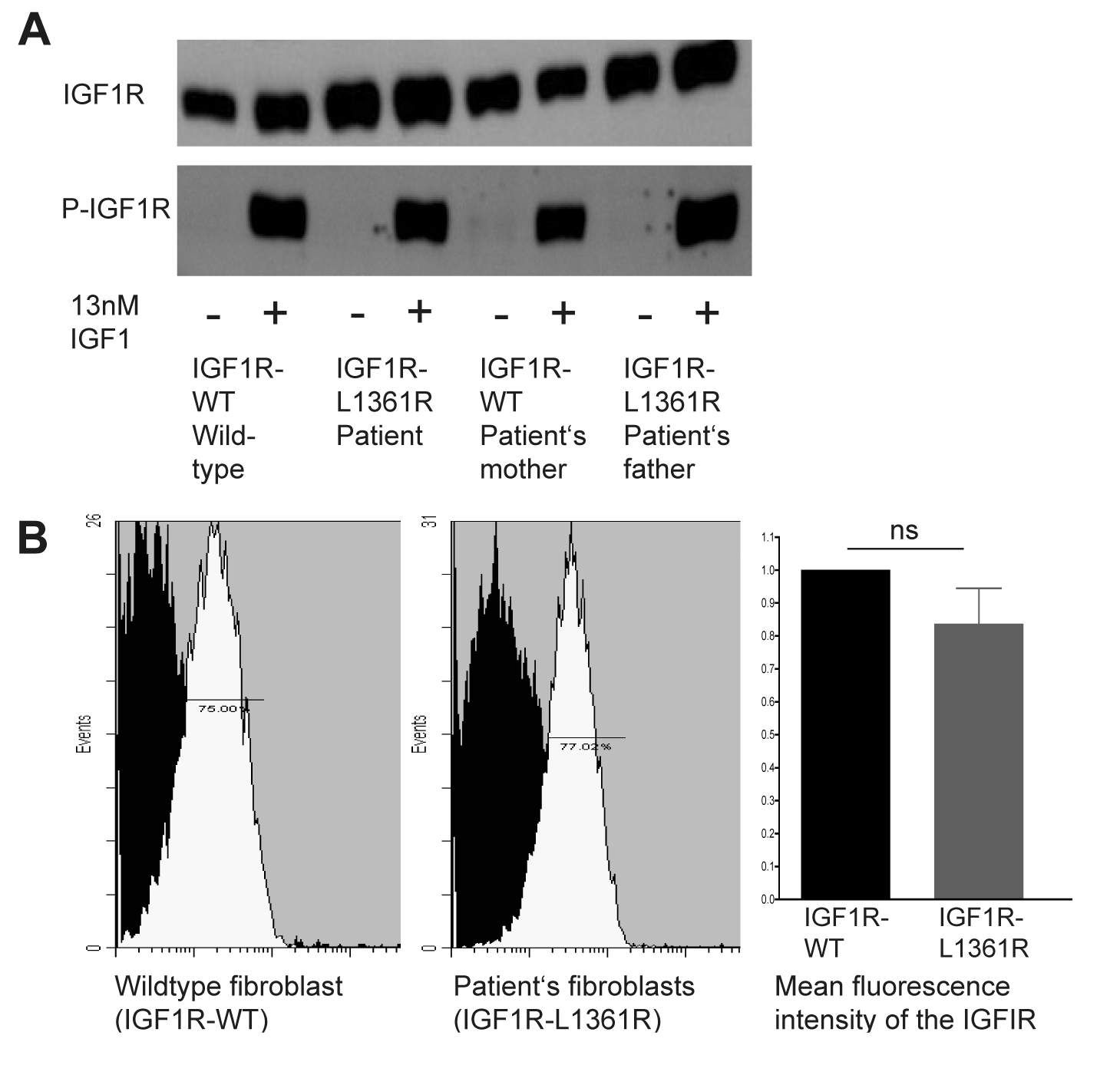

Supplement: Figure S2 — Protein and cell surface expression of IGF1R in wild type and patient's fibroblasts. A, Protein expression and autophosphorylation of IGF1R of wild type fibroblasts (IGF1R-WT), patient's fibroblasts (IGF1R-L1361R) and parental fibroblasts after stimulation with 13 nM (100 ng/ml) IGF1 for 15 minutes was assessed by immunoblotting. Blots were incubated with specific antibodies against phosphorylated IGF1R β-subunit (P-IGF1R), stripped and subsequently incubated with specific antibodies against IGF1R α-subunit (IGF1R). Immunoblots shown are representative for three independent experiments. B, Expression of the IGF1R on cell surface of wild type and patient's fibroblasts. Cells were stained with phycoerythrin (PE) labelled antibodies and analyzed by flow cytometry. Black curve marks cells labeled with isotype control PE antibody, white curve marks cells labeled with human anti-IGF1R-PE antibody. Percentages of IGF1R PE positive cells are indicated. The mean fluorescence intensity of the IGF1R-phycoerythrin-antibody positive cells normalized to wild type fibroblasts represent the amount of cell surface IGF1R. Results are shown as means ± SEM calculated from more than three independent experiments. (TIF) [file pone.0038220.s002.tif]
